# In Vitro Comparative Study of Emulsified Fat Grafts

**Published:** 2020-04-17

**Authors:** Raghda Fayek Shaker, Abdel Rahman M. Abdel Aal, Khaled Mohamed Ali El Gazzar, Fatma Abdel Karim Abu Zahra, Ahmed Elshahat

**Affiliations:** ^a^Plastic Surgery Department, Faculty of Medicine, Ain Shams University, Cairo, Egypt; ^b^Cell and Culture Unit, Medical Research Center, Faculty of Medicine, Ain Shams University, Cairo, Egypt

**Keywords:** nanofat, collagenase, lipofilling, fat injection, stem cells

## Abstract

**Introduction:** Fat grafting is considered one of the most precious armamentarium in the hand of plastic surgeons. The fat grafts consist of 2 components, adipocytes and stromal cells. The adipose tissue is a reserve of stem cells. **Aim:** The aim of this study was to compare the adipocyte and stem cell viability in both mechanically processed and enzymatically digested fats. **Patients and Methods:** This in vitro study was conducted using 40 specimens collected from 20 patients who underwent liposuction procedures. Twenty specimens were mechanically processed (group A), whereas the remaining specimens were processed enzymetically (group B). **Results:** There were no statistically significant differences between fat cell viability between the 2 groups. On the contrary, there was statistically significant increase in stem cells in mechanically processed fat specimens (*P* = .001). **Conclusion:** Both the mechanically and chemically processed fat techniques are reliable techniques that provide fat and stem cells. Mechanical processing is easier and provides more stem cells.

The first reports on autologous fat grafting were published in the early 20th century.^1^ The procedure became more widely implemented after Illouz^2^ introduced liposuction in the 1980s. Since the standardization of the fat grafting technique by Coleman^3^ a decade later, lipofilling has become a very important and valuable technique in plastic surgery.^4^ Since then, the use of fat grafting among surgeons and patients has markedly risen, as fat has many characteristics making it better than any synthetic filler, as it is natural, is obtained from the patient, is usually present in a good amount in most patients, and can be easily removed.^5^

Aspirated adipose tissue consists of 2 components, lipid-containing adipocytes and stromal cells containing cellular compartments.^6^ Adipose tissue is a reserve of mesenchymal stem cells that can divide indefinitely, producing various cellular lines.^7^ Adipose-derived stem cells are prevalent around blood vessels and within the connective tissue of human adipose tissue. This stromal vascular fraction (SVF) of adipose tissue can be isolated from either suction-aspirated adipose tissue or excised human fat by enzymatic collagenase digestion.^8^

Conventional fat grafting may leave visible lumps. Better results can be obtained by performing lipofilling with cannulas as small as 0.7 mm in diameter; this is also called microfat grafting.^9^

Mechanically processed fat produces more regular and finer particles (nanofat), unlike the microfat grafting.^10^^,^^11^ Chemically digested fat, using collagenase for digestion of the fat graft, creates more liquid filler for injection.^12^^,^^13^

To our knowledge, no previous study in literature has compared mechanically and chemically processed fats.

The aim of this study was to compare the viability and concentration of adipocytes and stem cells in both mechanically processed and enzymatically emulsified fats.

## PATIENTS AND METHODS

Patients who attended the plastic surgery department in the hospital were recruited to the study. The inclusion criterion for patients who volunteered to provide the fat samples was women between 18 and 50 years of age. This study comprised 20 patients; the fat sample was taken from the each participant and was divided into 2 samples, one sample for each group. The total samples taken were 40, with each group having 20 samples: group A mechanically processed fat and group B enzymatically digested fat.

Patients were thoroughly advised about the nature of the study, the treatment options, and possible complications, and informed consent was obtained. The study was conducted at the Medical Research Center of the hospital.

Fat was harvested from the lower abdomen or, in some cases, from the inner thigh, depending on the patient's natural fat deposits. We employed the tumescent technique, which consists of infiltration of the fat layer with a vasoconstrictive drug (diluted adrenaline 0.001%) in areas to be aspirated to lessen the bleeding. The technique of fat harvesting during liposuction was standardized using cannulas with a 3-mm inner diameter and a blunt tip. The harvested fat was centrifuged at 1500 rpm (with radius of 5 and 126 *g*) for 3 minutes to obtain purified fat using the Coleman method. Cellular debris at the bottom was removed, and the oily layer was removed using a sterile pad.

Next, the fat was divided into 2 groups to be processed differently. For group A, the lipoaspirate was mechanically processed by shifting the fat 50 times between 2 syringes connected by a female-to-female Luer Lock connector. The processed fatty liquid was filtered, and the effluent was collected in a sterile 15-mL Falcon tube. The aspirate was then washed with phosphate buffered saline (PBS) 6 times to remove all the debris. After washing, a sample of the fat was taken to count the number of cells under a microscope using a hemocytometer and to assess the viability of the fat cells.

Next, a trypan blue assay was performed to test adipocyte viability and detect the survival of fat cells. If the cells took up the trypan blue, they were considered nonviable. The surviving fat cells were then counted using the hemocytometer.

The remaining fat was then processed to obtain stem cells from mechanically processed fat by adding 0.15 mg of collagenase in 100 mL of PBS for every 3 mL of fat. This was then incubated in a water bath for 25 minutes at 37 °C and centrifuged at 800 rpm (with radius 10.7 and 77 *g*) for 3 minutes to remove impurities. After digestion, the suspension was neutralized with an equal volume of culture medium (Dulbecco's Modified Eagle Medium or DMEM), resulting in a pellet with a large number of adipose-derived stem cells. The stem cells were isolated from the fat graft and counted using the hemocytometer. The viability of the stem cells was also assessed. The total time needed for the procedure was about 30 minutes.

**Figure 1:**
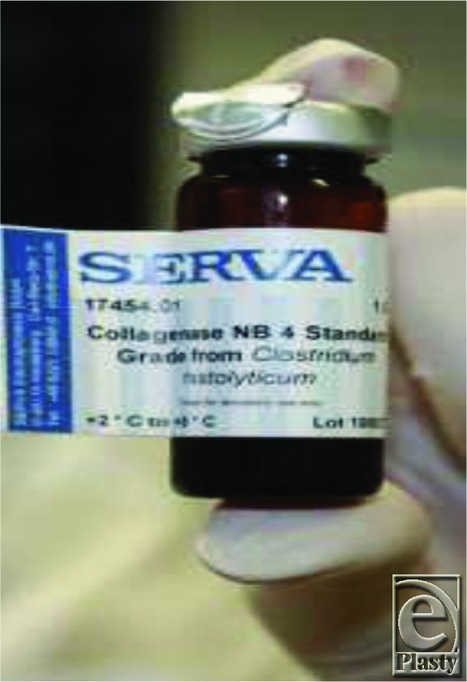
Collagenase type I vial.

For group B, the harvested fat was washed with PBS. The remaining fat layer was mixed with 0.15% *Clostridium histolyticum*–derived collagenase type I (Sigma-Aldrich, St Louis, Mo) (Fig 1) in 100 mL PBS for every 3 mL of fat. This was then incubated in a water bath for 25 minutes at 37 °C and centrifuged at 800 rpm (77 *g*) for 3 minutes to remove impurities. After digestion, the suspension was neutralized with an equal volume of culture medium (DMEM), resulting in a pellet with a large number of adipose-derived stem cells.

After washing, a sample of the fat was taken to count the number of cells under a microscope using a hemocytometer. The viability of the fat cells was also assessed. Another sample was taken to count the number of stem cells and to assess their viability.

Next, a trypan blue assay was performed to determine the survival of fat cells and their viability. If the cells took up the trypan blue, they were considered nonviable. The viable cells were not stained with the trypan blue. To calculate the number of viable cells per milliliter of the culture, we used the following formula: Number of viable cells × 10^4^ × Dilution factor = Viable cells/mL of culture). To determine the viability percentage, we used the following formula: Viability percentage = [Total viable cells (unstained)/Total cells] × 100.

The presence of stem cells was confirmed by the formation of cell pellet at the bottom of the Falcon tube. The supernatant was carefully removed by a pipette, leaving the pelleted SVF. The cell suspension was resuspended in a 10-mL complete culture comprising DMEM, 13% FBS (fetal bovine serum), and 1.5% penicillin streptomycin (Lonza, Verviers, Belgium).

**Figure 2:**
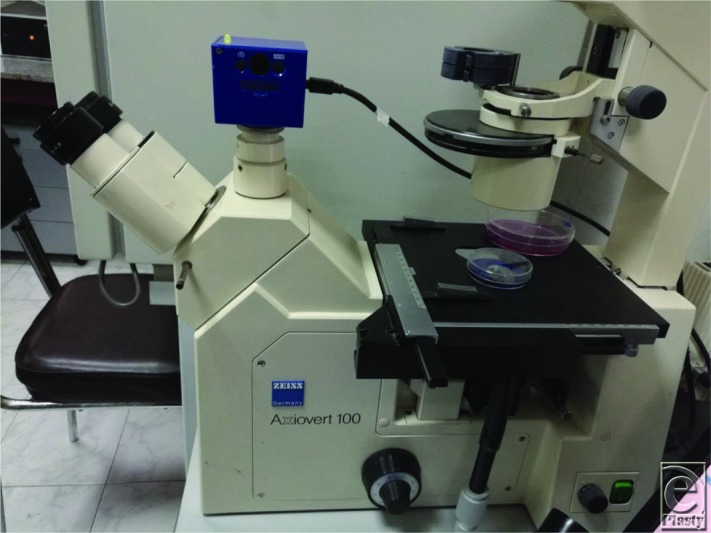
Inverted microscope (Axiovert 100; Zeiss, Jena, Germany).

**Figure 3:**
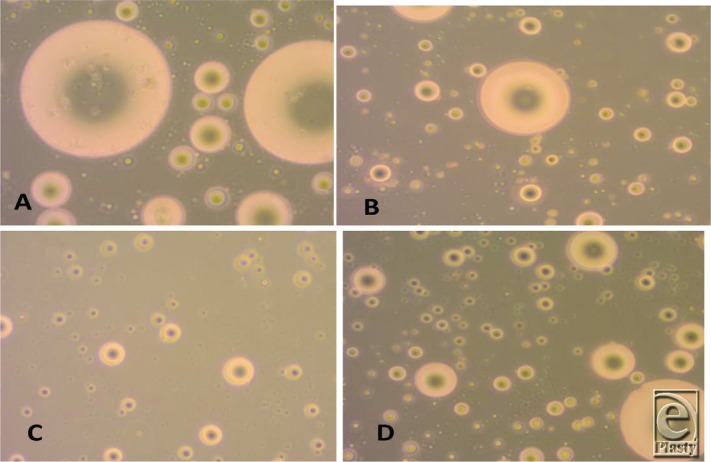
Cells after being examined by the inverted microscope: (*a*) fat cells mechanically digested, (*b*) stem cells mechanically digested, (*c*) fat cells enzymatically digested, and (*d*) stem cells enzymatically digested.

**Figure 4:**
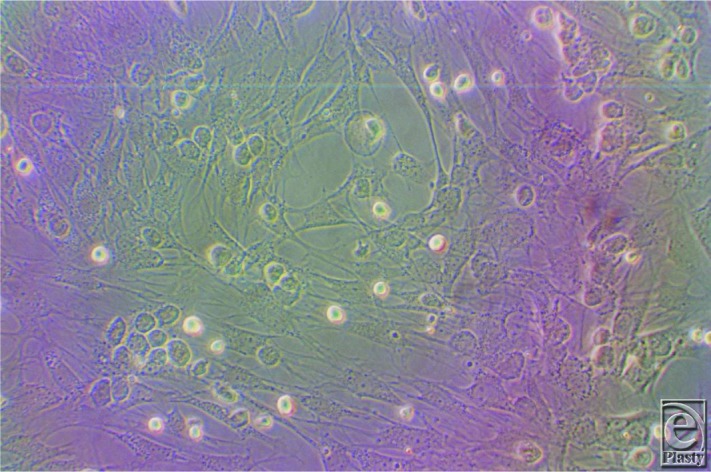
Mesenchymal stem cells with 80% confluence as seen by the inverted microscope.

The cell suspension was cultured in 25-cm culture flasks (Easy Flask; Nunc, Roskilde, Denmark) and incubated in a CO_2_ incubator (Nuaire NU 4950E, Autoflow Water Jacketed CO_2_ incubator; NuAire, Inc, Plymouth, Minnesota) at 37 °C with 5% CO_2_. Before culturing, the culture flasks were examined using an inverted microscope (Axiovert 100; Zeiss, Jena, Germany) (Fig 2). The medium was replaced every 3 days, the nonadherent cells were discarded, and the attached cells were washed with PBS. The expansion of adipose stem cells (ASCs) was followed by an examination using an inverted microscope (Figs 3 and 4).

When the cells reached confluence at day 12 of culturing (confluent cells consisted of a dense homogeneous population of spindle-shaped cells), trypsinization was performed using 0.25% trypsin-EDTA solution (Lonza) to detach the cells from the culture flasks. After 5 minutes, the cells were examined to confirm their detachment. We then confirmed mesenchymal stem cell differentiation into osteoblasts and neural cells.

The identification of ASC surface markers was performed by incubating cell aliquots (1 × 10^6^ cells) in the dark for 30 minutes at room temperature with fluorescein isothiocyanate–conjugated monoclonal antibodies against CD44 (Beckman Coulter, Clare, Ireland) and phycoerythrin-conjugated monoclonal antibodies against CD105 (Beckman Coulter). (For the optimal performance of fluorochrome-conjugated antibodies, the samples were protected from light.)

The cells were washed by adding 1 mL of PBS to wash off excess antibodies and then centrifuged at 1200 rpm for 3 minutes. The supernatant was removed, and the pellet was resuspended in 1 mL of PBS. Next, the cells were examined using a Navios flow cytometer (Beckman Coulter), and the data were analyzed using Navios software version 1.2 (Beckman Coulter) and expressed in the form of a histogram showing the percentage of positive cells. The total time for this procedure was about 4 hours.

## RESULTS

**Figure 5:**
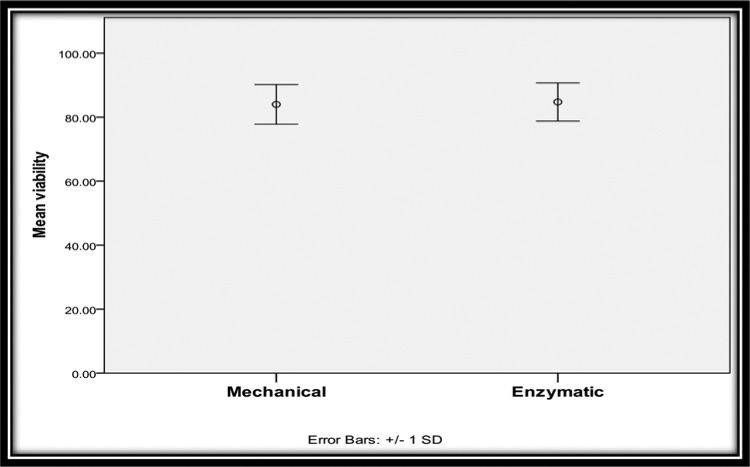
Comparison between 2 groups regarding the percentage of viability.

All the data were collected and tabulated according to cell viability or the number of fat cells and stem cells in both types of fat grafts using the hemocytometer. A statistical analysis was completed using independent-samples *t* tests. For group A, the results showed the viability of cells in mechanically processed fat, with a mean of 84.00 and a standard deviation (SD) of 6.20. For group B, the results showed the viability of cells in enzymatic digested fat, with a mean of 84.75 and an SD of 5.95. There was no significant difference between the groups (Table 1; Fig 5).

**Figure 6:**
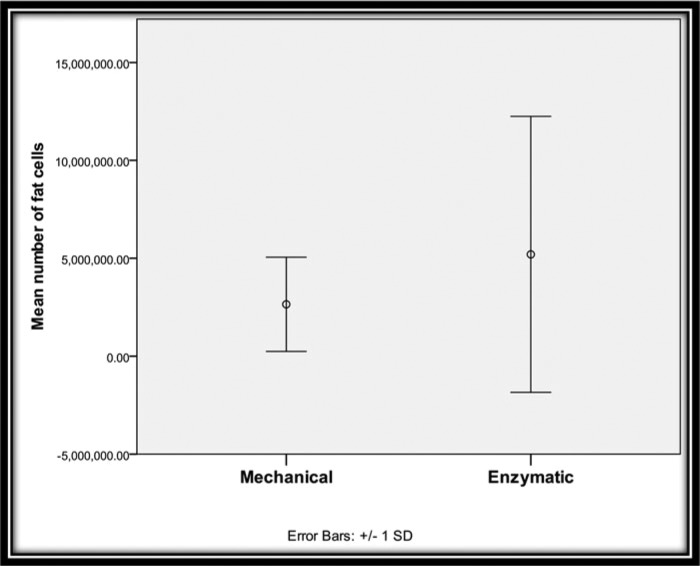
Comparison between 2 groups regarding the number of fat cells.

Regarding the number of fat cells, group A showed a mean of 2,650,000.00 and an SD of 2,405,419.32 whereas group B showed a mean of 5,199,627.25 and an SD of 7,044,952.98. There appears to be no statistically significant difference between the groups (Table 2; Fig 6).

**Figure 7:**
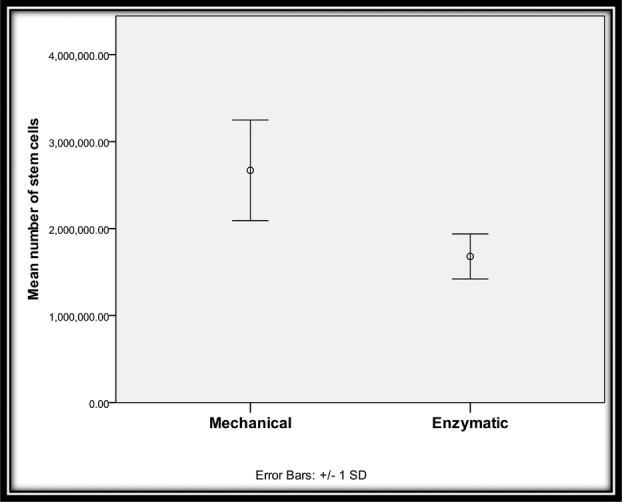
Comparison between 2 groups regarding the number of stem cells.

Regarding the number of stem cells, the stem cells in mechanically processed fat, with a mean of 2,670,000.00 and an SD of 578,255.09, were greater in number than the stem cells in enzymatically digested fat with a mean of 1,680,000.00 and an SD 258,660.52. This is statistically significant difference (Table 3; Fig 7).

**Figure 8:**
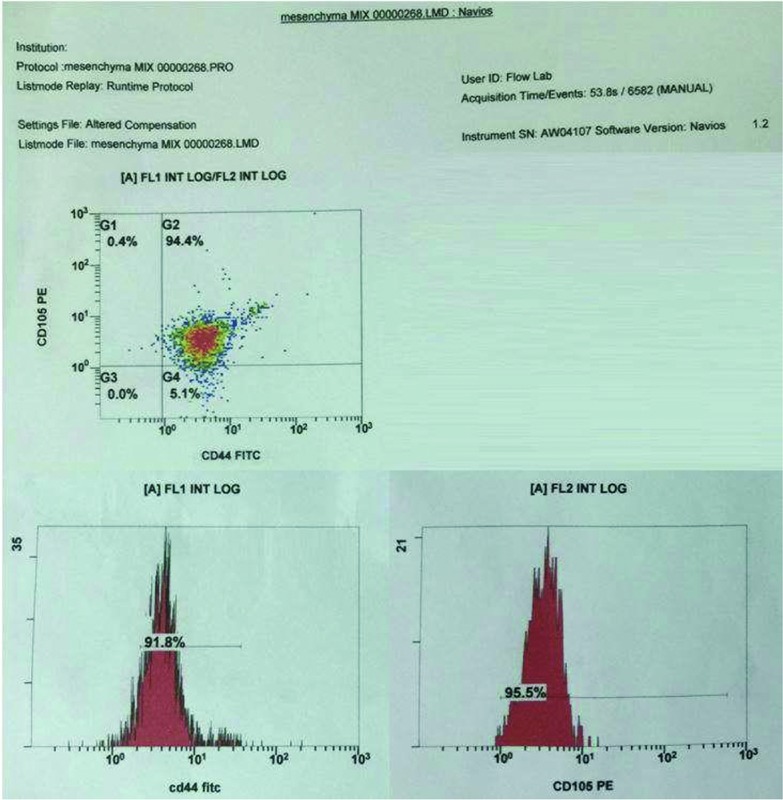
Histogram of flow cytometry analysis showing stem cell expression of CD44 and CD105.

An examination of the stem cell pellet revealed and ensured the required characterization using flow cytometry analysis. The results showed that within passage 0, stem cells were positive for CD44 (91.8%) and CD105 (95.5%) (Fig 8). These results are consistent with the characterization of stem cells.

## DISCUSSION

Tonnard et al^10^ were considered the first to have discussed and used mechanically processed fat as a superficial fat graft. Many others have since used the same technique. We used this technique in the present study and compared it with the use of collagenase-digested fat, which has been studied by many authors, such as Moscatello et al^13^ in 2008, Seungki et al^14^ in 2013, and Shoukralla et al^15^ in 2014. The present study is the first study in the literature to discuss and compare both techniques, giving comparable and reliable results for both techniques in terms of the number and viability of adipocytes and stem cells.

Different factors have been discussed in many studies concerning fat graft survival. Two such factors are the negative pressure applied and the size of the cannula used in harvesting fat. In a 2001 study, Asken^16^ found that 90% of fat extracted by liposuction appears viable, assuming it is not traumatized either by handling or by high-suction pressure. Damage to the adipocytes is inversely related to the diameter of the instrument used for harvesting and the injection.

In the present study, the fat was suctioned using the tumescent technique and a 3-mm cannula under moderate negative pressure generated by the syringe. We found that there is a very good count of viable adipocytes and stem cells.

Some authors previously discussed the effect of trauma and dissection on fat viability. In 1938, Guerney^17^ stated that crushed grafts eventually disappear, attesting to the devastating effect of trauma on the viability of a graft.

Agris^18^ found that the nanofat affects the dermis thickness, as histological analysis revealed that nanofat had increased dermal thickness and good collagen fiber arrangement with high capillary density and cell proliferation due to the presence of a high amount of stem cell content. This is confirmed in our study by a high count of stem cell content in the specimens.

The mechanical processing of fatty samples leads to division of the large clusters of fat into small viable clusters and does not affect the viability of fat and stem cells unlike the chemical digestion with the collagenase enzymatic method, as this method leads to chemical dissolution of fat clusters that affects the viability and number of fatty cells as well as stem cells. This can explain and clarify the results of the current study as regard to the viability and number of fat cells and stem cells.

In this current study, we used the mechanical method and compared it with the collagenase method. It was found that the mechanical method for preparation of samples took about 30 minutes, which is a short period of time to prepare the specimens. Also, there is no need for complex and sophisticated expensive equipment and materials as in the collagenase method, which, on the contrary, takes about 4 hours to prepare specimens, which is a longer time than that required in the mechanical method and complex, sophisticated, and expensive equipment and materials are needed in addition to the need for a well-trained person to prepare the specimens as well as to deal with the machine.

The results of the current study are limited, as it is an experimental work and there is a need for further clinical study and different clinical applications.

Informed consent was obtained from all patients in order to use the lipoaspirate for analysis. This was applicable for the mechanical emulsified fat as well as collagenase-digested fat. Also, the current study was approved by the ethical committee of the Faculty of Medicine, Ain Shams University.

## CONCLUSION

Mechanically processed fat method has many advantages over the enzymatic method, as it is easy, less time-consuming, and reliable. It gives a high content of fat viable cells and stem cells.

## Figures and Tables

**Table 1:** Comparison between 2 groups regarding the percentage of viability*

	Percentage of viability					
	Min	Max	Mean	SD	*t*^†^	*P*
Mechanical	70.00	90.00	84.00	6.20	0.39	.70
Enzymatic	70.00	90.00	84.75	5.95		NS

*NS indicates not significant.

^†^Independent-samples *t* test.

**Table 2:** Comparison between 2 groups regarding the number of fat cells*

	Number of fat cells					
	Min	Max	Mean	SD	*t*^†^	*P*
Mechanical	1,260,000	12,000,000	2,650,000.00	2,405,419.32	1.53	.14
Enzymatic	2,000,000	32,000,000	5,199,627.25	7,044,952.98		NS

*NS indicates not significant.

^†^Independent-samples *t* test.

**Table 3:** Comparison between 2 groups regarding the number of stem cells*

	Number of stem cells					
	Min	Max	Mean	SD	*t*^†^	*P*
Mechanical	780,000	3,360,000	2,670,000.00	578,255.09	6.99	<.001
Enzymatic	1,220,000	2,400,000	1,680,000.00	258,660.52		HS

*HS indicates highly significant.

^†^Independent-samples *t* test.
